# Aldehyde Dehydrogenase 2 as a Therapeutic Target in Oxidative Stress-Related Diseases: Post-Translational Modifications Deserve More Attention

**DOI:** 10.3390/ijms23052682

**Published:** 2022-02-28

**Authors:** Jie Gao, Yue Hao, Xiangshu Piao, Xianhong Gu

**Affiliations:** 1 State Key Laboratory of Animal Nutrition, Institute of Animal Sciences, Chinese Academy of Agricultural Sciences, Beijing 100193, China; gaojieludou@126.com (J.G.); haoyueemail@163.com (Y.H.); 2 State Key Laboratory of Animal Nutrition, College of Animal Science and Technology, China Agricultural University, Beijing 100193, China; piaoxsh@cau.edu.cn

**Keywords:** aldehyde dehydrogenase 2 (ALDH2), post-translational modifications (PTMs), oxidative stress-related diseases, 4-hydroxy-2-nonenal (4-HNE)

## Abstract

Aldehyde dehydrogenase 2 (ALDH2) has both dehydrogenase and esterase activity; its dehydrogenase activity is closely related to the metabolism of aldehydes produced under oxidative stress (OS). In this review, we recapitulate the enzyme activity of ALDH2 in combination with its protein structure, summarize and show the main mechanisms of ALDH2 participating in metabolism of aldehydes in vivo as comprehensively as possible; we also integrate the key regulatory mechanisms of ALDH2 participating in a variety of physiological and pathological processes related to OS, including tissue and organ fibrosis, apoptosis, aging, and nerve injury-related diseases. On this basis, the regulatory effects and application prospects of activators, inhibitors, and protein post-translational modifications (PTMs, such as phosphorylation, acetylation, S-nitrosylation, nitration, ubiquitination, and glycosylation) on ALDH2 are discussed and prospected. Herein, we aimed to lay a foundation for further research into the mechanism of ALDH2 in oxidative stress-related disease and provide a basis for better use of the ALDH2 function in research and the clinic.

## 1. Introduction

Oxidative stress (OS) plays a key role in the pathogeneses of various etiologies. Reactive oxygen species (ROS) are mainly produced from the mitochondrial electron transport chain and enzymatic systems, such as xanthine oxidase and reduced nicotinamide adenine dinucleotide (NADH)/reduced nicotinamide adenine dinucleotide phosphate (NADPH) oxidase [1,2]. Under harmful stimulation, the production and accumulation of ROS exceed the ability of cells to remove them; this leads to oxidant–antioxidant imbalance and induces OS, resulting in oxidative attack on biological macromolecules that causes cell damage [3,4]. Membrane lipids are one of the most vulnerable targets of ROS damage [5], and increased ROS triggers lipid peroxidation. The main primary products of lipid peroxidation process are lipid hydroperoxides (LOOH), and the secondary products contain more than 200 types of highly active lipid-derived aldehydes (LDAs) [6], which can further aggravate cell damage [5]. Some aldehydes are strongly electrophilic, highly reactive, and toxic, including 4-hydroxy-2-nonenal (4-HNE), malondialdehyde (MDA), acrolein, 4-oxo-2-nonenal (4-ONE), and crotonaldehyde [5,7]. These LDAs further mediate the biological effects of free radicals, leading to the sustained generation of mitochondrial ROS and the consequent aggravation of tissue damage, thus amplifying the OS [8,9]. Reactive aldehydes easily form adducts with proteins, deoxyribonucleic acid (DNA), and phospholipids, inducing mutations in critical genes, the inactivation of key enzymes and consequent cell damage, and OS-related diseases such as cardiopathy, vasculopathy, cancer, diabetes, osteoporosis, and neurodegenerative diseases (NDDs) [10,11,12,13,14].

The aldehyde dehydrogenase (ALDH) family plays important roles in the metabolism of aldehydes in vivo. ALDH2 is an ALDH family member that catalyzes the oxidation of various aldehydes; it is widely distributed throughout the body as an oxidoreductase, but is mainly found in the liver, heart, lungs, kidneys, and other mitochondria-rich tissues. ALDH2 can remove aldehydes produced during cellular-substance metabolism and OS to reduce cytotoxicity and oxidative damage. ALDH2 can be activated by several pathways, its gene polymorphism and activity are related to a variety of pathophysiological processes, and it is widely involved in and affects the occurrence and development of various diseases and important metabolic processes. Consequently, it is attracting increasingly more attention as a key regulatory enzyme in OS. The structure and function of ALDH2, as well as the regulation of its dehydrogenase activity, are the subjects of this review.

## 2. ALDH2 in Ethanol and Aldehyde Metabolism

### 2.1. Human ALDH Protein Family

ALDH proteins, a class of nicotinamide adenine dinucleotide (phosphate) (NAD[P]^+^)-dependent enzymes [15,16], are mainly localized in subcellular compartments such as the cytoplasm, mitochondria, the endoplasmic reticulum (ER), and nuclei. They have common structural and functional characteristics (Table 1); they can catalyze the oxidation of various aliphatic and aromatic aldehydes, and can participate in the oxidation of many endogenous and exogenous aldehydes into corresponding carboxylic acids [15,16,17] to protect cells from oxidative damage caused by the accumulation of aldehydes during metabolism [18]. So far, the human genome has been found to contain 19 putatively functional *ALDH* genes (Table 1), which are located on different chromosomes and have different substrates and reactivities.

### 2.2. Effect of ALDH2 on Detoxification of Reactive Aldehyde

ALDH2 is the ALDH with the highest affinity (Km < 5 μM) for acetaldehyde [16,65], and has catalytic activity for various toxic aldehydes. The main toxic effect of aldehydes results from covalent modifications of several biological macromolecules, which produce adducts that interfere with the physiological functions of these biological macromolecules [66] and lead to protein inactivation and DNA damage. Reactive aldehydes can bind to nucleophilic amino acids (i.e., cysteine, histidine, and lysine), bases in nucleic acids, and lipids with amino groups through Michael addition or Schiff base formation [67,68,69], which in turn damages the macromolecules and impairs cellular functions. The C3 of C2=C3 double bond of 4-HNE can target thiol or amino groups of proteins by Michael additions, and the C1 carbonyl groups of 4-HNE react with primary amines of proteins to yield Schiff bases [5,67]; The Michael addition of a C=C double bond of 4-HNE to the NH_2_-group of deoxyguanosine also yields exocyclic adduct [5,67]. Similarly, MDA reacts with guanine, adenine, and cytosine to form adducts of deoxyguanosine and deoxyadenosine; then, exocyclic DNA adducts that are produced lead to DNA damage and mutation [68].

In addition, the mutual triggering of aldehydes and ROS (mainly refers to hydroxyl radical [HO^•^] and hydroperoxyl [HO_2_^•^]) aggravates damage to cellular components, *viz.*, lipids, proteins, and nucleic acids [5]. On the one hand, ROS-triggered lipid oxidation is the most common source of toxic aldehydes, such as 4-HNE [67]; OS induced by ROS accumulation causes lipid peroxidation and the production of active aldehydes, which can interact with sulfhydryl and amino groups to eliminate endogenous antioxidants, such as glutathione [70,71]. On the other hand, the NADH/NADPH oxidase system is a major source of ROS [1], and the promoting effect of 4-HNE on ROS could be related to its regulation of the NADH/NADPH enzyme system. Under physiological conditions, low concentrations of aldehydes can induce antioxidant defense [2,72], but when the aldehydes accumulate past a certain threshold, they will continue to trigger the production of ROS. The oxidation process of ethanol and aldehydes increases the NADH/NAD^+^ ratio in both the cytosol and mitochondria, which improves xanthine oxidase activity and catalyzes the generation of superoxide free radicals [13,72]. 4-NHE was also reported to promote NADPH oxidase activity through increased membrane translocation of p47phox (subunit of NADPH oxidase) with subsequent enhancement of the production of ROS [73]. 4-HNE is capable of inducing mitochondrial ROS production through a mechanism that requires activities of NADH-ubiquinone oxidoreductase (Complex I) of the electron transport chain [71]. ALDH2 can decompose the highly toxic aldehydes produced during lipid peroxidation into non-toxic carboxylic acids [16], which is beneficial for reducing aldehyde-induced tissue damage [66,74,75] and protecting cells from OS [18]. Therefore, ALDH2 is a major protective enzyme.

**Figure 1:**
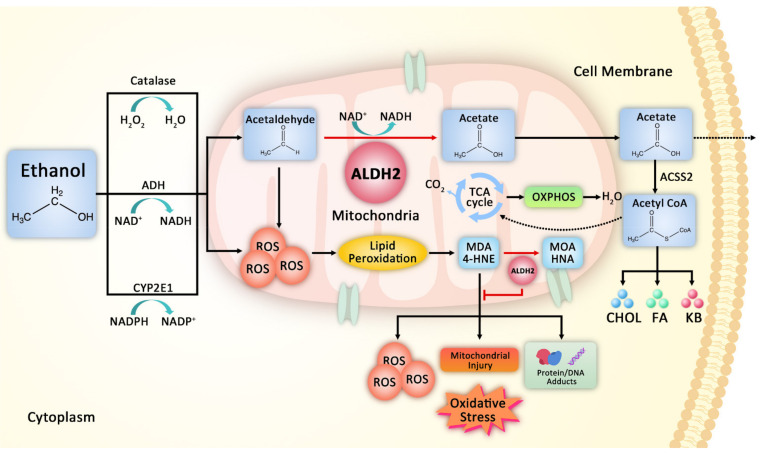
Pathway by which aldehyde dehydrogenase 2 (ALDH2) catalyzes aldehyde metabolism in ethanol metabolism. ALDH2 metabolizes acetaldehyde into acetate, metabolizes the malondialdehyde (MDA) into malonic acid (MOA) or acetaldehyde, and metabolizes the 4-hydroxy-2-nonenal (4-NHE) to generate 4-hydroxy-2-nonenoic acid (NHA) to reduce the accumulation of toxic aldehydes produced during metabolism. CHOL = cholesterol; FA = fatty acids; KB = ketone bodies.

ALDH2 plays an important role in the metabolism of toxic aldehydes directly and indirectly produced by ethanol metabolism (Figure 1). Ethanol is oxidized to acetaldehyde by three main pathways: (1) the vast majority of ethanol is oxidized by alcohol dehydrogenases (ADHs) in cytoplasm under the action of NAD^+^; (2) some of the ethanol is oxidized via the microsomal ethanol-oxidizing system (MEOS), which relies on cytochrome P4502E1 (CYP2E1); and (3) a small amount of ethanol is converted into acetaldehyde under the action of catalase (CAT) [76]. ALDH2 further metabolizes acetaldehyde into acetate, which can be transported out of the cell through a carrier or converted into acetyl-coenzyme A (acetyl-CoA) through the enzymatic action of cytosolic acetyl-CoA synthetase 2 (ACSS2) [66]. Then, acetyl-CoA enters different physiological pathways, including energy metabolism pathways such as the tricarboxylic acid (TCA) cycle or the oxidative phosphorylation (OXPHOS) pathway [77]. Eventually, it is metabolized into water and carbon dioxide. Acetyl-CoA also participates in the production of ketone bodies (KB), fatty acids (FA), and cholesterol (CHOL) [66,78]. In addition, the MDA produced in lipid peroxidation is converted into malonic semialdehyde under the action of ALDH2 and further generates malonic acid (MOA), or alternatively, a carboxyl group is removed and converted into acetaldehyde; ultimately, acetyl-CoA is generated and enters the TCA cycle [79]. Some of the 4-NHE produced during lipid peroxidation generates 4-hydroxy-2-nonenoic acid (NHA) under the action of ALDH2, while the remainder is catalyzed by other reductase enzymes such as glutathione-S-transferase (GST), aldo-keto reductases (AKRs)/ADHs into s glutathionyl-HNE (GS-HNE), and 1,4-dihydroxy-2-nonene (DHN) [80,81]. Loss or inhibition of ALDH2 biological activity interferes with its detoxification effect on aldehydes, which in turn triggers the production of ROS and amplifies oxidative damage to cells and leads to a variety of human diseases [75,82].

## 3. Structure and Gene Polymorphism of ALDH2

**Figure 2:**
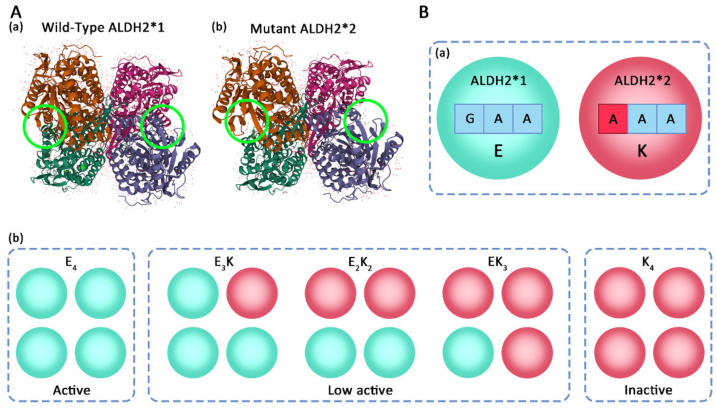
Tetramer structure of ALDH2. (**A**) (**a**) Tetramer structure of wild-type (WT) ALDH2 (ALDH2*1, Protein Data Bank [PDB] Accession Code: 1O05). (**A**) (**b**) Tetramer structure of the inactive mutant of ALDH2 (ALDH2*2, PDB Accession Code: 1ZUM). Green circle: the α-helix structure missing from the mutant tetramer versus WT; modified from [105]. (**B**) (**a**) “E” = subunit encoded by the WT allele ALDH2*1; the first base of codon 487 is guanine (G), and the 487th amino acid residue is glutamic acid (Glu/E). “K” = subunit encoded by the mutant allele ALDH2*2; the first base of codon 487 is mutated to adenine (A), and the 487th amino acid residue is changed from Glu to lysine (Lys/K). (**B**) (**b**) E_4_ homotetramers reflect normal enzyme activity; heterologous tetramer E_3_K, E_2_K_2_, and EK_3_ enzyme activities are substantially reduced; and K_4_ homotetramer enzyme activities are almost lost.

The *ALDH2* gene was first identified in 1987 [83], and its X-ray crystal structure was completely analyzed in 1999 [84,85]. The gene encoding human ALDH2 is located on the long arm of chromosome 12 (12q24.2) and contains 13 exons. It encodes the polypeptide chain of the enzyme precursor protein, which consists of 517 amino acid residues; the first 17 amino acids are mitochondrial guide peptides, which are cut after they help the enzyme enter the mitochondrial matrix to obtain mature enzyme polypeptide [17,65,85,86]. The analysis of the protein structure of ALDH2 shows that the enzyme is a tetramer composed of four identical subunits, each of which consists of three main domains with different functions: catalytic, NAD^+^ coenzyme-binding, and oligomerization [12,87,88]. Glu487 forms hydrogen bonds with Arg264 residues of the same subunit in ALDH2 holoenzyme, and forms hydrogen bonds with Arg475 of the adjacent subunit through hydrogen bonds to form dimers, and the two dimers assemble into a tetramer [89,90]. The catalytic mechanism of ALDH2 is that NAD^+^ first binds to ALDH2 by multiple binding sites to activate the key active-site cysteine residue (Cys302). The sulfhydryl group of Cys302 has high nucleophilicity and conducts nucleophilic attacks on the carbonyl carbon of the substrate aldehyde to form a thiohemiacetal intermediate. Substrate hydride is transferred from the thiohemiacetal intermediate to the nicotinamide ring of NAD^+^ to form NADH, which is accompanied by the production of thioester intermediates. Then, the structure of the enzyme-substrate complex changes [89,91]; the adjacent glutamate residue Glu268 activates H_2_O to remove protons from Cys302, and the carbonyl carbon of the thioester intermediate undergoes nucleophilic attack, resulting in the destruction of the carbon–sulfur bond to regenerate the free enzyme. The final product, carboxylic acid, is obtained, followed by NADH release, completing the reaction [92,93,94]. Guanine at the 1510^th^ nucleotide position (from the initiation codon) in exon 12 of the human *ALDH2* gene is replaced by adenine (G → A), which changes codon 487 from GAA to AAA, leading to a glutamic to lysine amino acid change (Glu → Lys) [65,95]. The Glu487Lys SNP of *ALDH2* (also known as Glu504Lys, rs671, c.1510G > A, p.E504K) [65,96] changes the wild-type (WT) allele ALDH2*1 into the mutant allele ALDH2*2, resulting in a decrease or loss of catalytic activity of the ALDH2 tetrameric enzyme (Figure 2B) [97]. As a result, three genotypes are produced: WT homozygous ALDH2*1/1, mutant heterozygous ALDH2*1/2, and mutant homozygous ALDH2*2/2 [98,99,100]. An *ALDH2* rs671 mutant is found in 30–50% of East Asians, while the ALDH2*2 variant is essentially absent in people of European descent (<5%) [101,102].

Glu487 is located within the oligomerization domain at the dimer interface of the tetrameric enzyme; this location is critical for the formation of both dimer and tetramer. Glutamic acid is mainly negatively charged at physiological pH, while lysine is mostly positively charged. The structures of the mutant subunit and its dimer chaperone are altered once Glu487 is replaced by Lys487 [103], a mutation that causes disorder of the α-helices (Figure 2A). The normal formation of α-helices near the dimer interface of each subunit is inhibited, which affects the formation of local secondary ALDH2 tetramer structures [12,88,104]. These changes cause the catalytic and coenzyme-binding domains of a single subunit to rotate away from the interface to destroy the NAD^+^ binding sites, resulting in severe loss of ALDH2 enzyme activity [88].

## 4. ALDH2 in Oxidative Stress-Related Physiological and Pathological Processes

**Figure 3:**
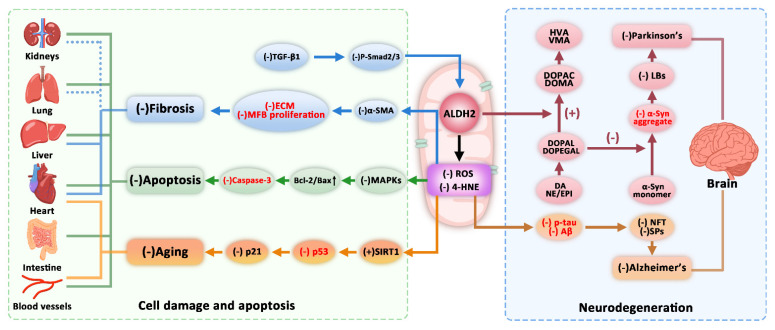
The main regulatory mechanism of aldehyde dehydrogenase 2 (ALDH2) in certain OS-related physiological and pathological processes. ALDH2 activation inhibits the transformation of human cardiac fibroblasts (HCFs) into myofibroblasts via the transforming growth factor-β1 (TGF-β)/*Drosophila* mothers against decapentaplegic protein (Smad) signaling pathway to inhibit fibrosis; ALDH2 inhibits mitogen-activated protein kinase (MAPK) signaling pathways and upregulates the B-cell lymphoma-2 (Bcl-2)/Bcl-2–associated X protein (Bax) ratio to reduce the cysteinyl aspartate–specific proteinase 3 (caspase-3) level and inhibit apoptosis; ALDH2 can also inactivate inhibit 4-hydroxy-2-nonenal (4-HNE) accumulation and regulate sirtuin 1 (SIRT1)/p53-dependent aging; ALDH2 can eliminate the toxic metabolites of neurotransmitters (dopamine [DA], epinephrine [EPI], and norepinephrine [NE]) and can inhibit α-Synuclein (α-Syn) abnormal aggregation as well as Lewy bodies (LBs) formation, and reduce neurofibrillary tangles (NFTs) formed by hyperphosphorylated microtubule-associated protein tau, so as to protect neurons from the damage caused by 4-HNE and reactive oxygen species (ROS). (+) and (−) mainly reflect upregulation/activation or downregulation/inhibition, respectively, related to ALDH2 function. Red = plays a key role in the realization of corresponding physiological/pathological processes.

The metabolism of various endobiotic and xenobiotic compounds produces a series of aldehydes. Endogenous aldehydes are formed when amino acids, carbohydrates, lipids, biogenic amines, vitamins, and steroids are metabolized [18,106,107]. In addition, the biotransformation of many drugs and environmental chemicals produces aldehydes [105]. Accumulation of these aldehydes in cells enhances the damage caused by OS and induces related diseases. As ALDH2 is an oxidoreductase, its catalytic deficiency is related to fibrosis, apoptosis, aging, and neurodegeneration (Figure 3) [108]. Epidemiological and functional studies have reported that ALDH2 Glu487Lys gene polymorphism and the alterations of its enzyme activity not only are related to adverse reactions to alcohol after drinking, such as severe facial flushing, nausea, headache, and tachycardia, but also play important roles in cardio- and cerebrovascular diseases (CCVDs), cancer, NDDs, osteoporosis and drug toxicity (Table 2, Figure 3) [109,110].

### 4.1. Fibrosis

The process of lipid peroxidation caused by OS produces aldehydes, which is related to tissue and organ fibrosis. Fibrosis is a common feature of most chronic diseases involving tissue damage [123], affecting multiple organs, such as the liver, kidneys, cardiovascular (CV) system, lungs, and skin. There are different harmful compounds and inducements that lead to the occurrence and development of fibrosis in different organs. The fibrotic response is characterized by an increase in extracellular matrix (ECM) components [124,125], such as fibronectin (FN), collagen, laminin, elastin, fibrillin, and proteoglycans, together with the proliferation, migration, and accumulation of mesenchymal cells [126]. Transforming growth factor-β (TGF-β) can regulate the migration, proliferation, and differentiation of endothelial cells, epithelial cells (ECs), and fibroblasts; promote the production of collagen and FN in ECs and mesenchymal cells; and lead to net accumulation of ECM during wound healing and fibrosis [127]. Increasing evidence shows that TGF-β acts as a key regulator of fibrosis.

ALDH2 plays a protective role in myocardial, liver, and other organ fibrosis caused by OS-related diseases and exposure to toxic chemicals [128]. Under the action of TGF-β, both *Drosophila* mothers against decapentaplegic protein 2 (Smad2) and Smad3 are activated by phosphorylation, and Smad2/3 is an upstream regulator of ALDH2. The activation of ALDH2 regulated by the TGF-β1/Smad signaling pathway inhibits the transformation of human cardiac fibroblasts (HCFs) into myofibroblasts, which is manifested by a decrease in α-smooth muscle actin (α-SMA) and periostin expression, as well as myofibroblastic proliferation, collagen formation, and contractility reduction [111]. Studies have shown that activation of ALDH2 can reduce expression of collagen types I and III (COL1, COL3) as well as α-SMA in rat hearts and reduce levels of β-catenin, phosphor–glycogen synthase kinase-3β (p-GSK-3β), and wingless-type mouse mammary tumor virus (MMTV) integration site family member 1 (*Wnt-1*), indicating that ALDH2 protects against myocardial infarction (MI)-related cardiac fibrosis by downregulating the *Wnt*/catenin signaling pathway [129]. In addition, ALDH2 is important in the pathogenesis of alcoholic cirrhosis. Feeding ethanol plus carbon tetrachloride (CCl_4_) to *ALDH2* knockout (KO) mice leads to the accumulation of malondialdehyde–acetaldehyde adducts (MAAs) in the liver, stimulating cells to produce pro-inflammatory cytokines, such as interleukin-6 (IL-6). Hepatic expression levels of α-SMA, TGF-β, and collagen type I alpha 1 (COLIA1) messenger ribonucleic acid (mRNA) have been shown to be much higher in *ALDH2*^−/−^ mice than in WT mice, which led to inflammation and accelerated liver fibrosis; this indicated that ALDH2 deficiency can aggravate liver inflammation and fibrosis in mice [114]. N-(1,3-Benzodioxol-5-ylmethyl)-2,6-dichlorobenzamide (Alda-1) can activate the nuclear factor erythroid 2-related factor 2/heme oxygenase-1 (Nrf2/HO-1) antioxidant pathway and mitosis regulated by Parkin protein to reduce CCl_4_-induced chronic liver fibrosis in *ALDH2* KO mice [115]. Numerous studies have suggested that the inhibition of the TGF-β/Smad pathway and the production of ROS stimulated by TGF-β exerts an anti-fibrotic effect on pulmonary and kidney fibroses. Therefore, we speculated that inhibition of TGF-β/Smad signaling can reduce collagen and α-SMA expression by activating ALDH2 to delay OS-related fibrosis in the heart, liver, and other organs [130,131,132,133,134,135,136,137]. However, these hypotheses need to be confirmed by additional relevant studies.

### 4.2. Apoptosis

ROS produced during OS can induce and regulate apoptosis. Apoptosis (programmed cell death) is a programmed physiological process that removes superfluous, damaged, or altered cells to regulate homeostasis in the organism. Excessive apoptosis can lead to a decline in tissue and organ function and is implicated in a growing number of diseases, whereas insufficient apoptosis leads to carcinogenesis [138]. Apoptosis is a complex protease cascade reaction process mediated by members of the cysteinyl aspartate-specific proteinase (caspase) family [139]. The upstream caspases activate caspase-9 and further activate downstream caspases-3 and -7 under the action of apoptosis-inducing factor (AIF), hydrolyzing target substances in cells on a large scale and degrading intracellular proteins to induce cell death [140]. B-cell lymphoma-2 (Bcl-2) family members are important regulators of apoptosis; studies have demonstrated that balance of the expression levels of anti-apoptotic Bcl-2 and pro-apoptotic Bcl-2–associated X protein (Bax) play a major role in the regulation of apoptosis, and the Bcl-2/Bax ratio represents the degree of apoptosis [113]. Activation of mitogen-activated protein kinases (MAPKs), including extracellular signal-regulated kinase 1/2 (ERK1/2), p38 mitogen-activated protein kinase (p38 MAPK), and c-Jun NH_2_-terminal kinase (JNK), are closely related to apoptosis induced by OS. It has been found that the activation of p38 MAPK and JNK exert pro-apoptotic functions [141,142]. The expression of Bcl-2 is regulated by the upstream JNK and p38 MAPK under specific treatments. The activation of JNK can reduce Bcl-2 protein levels, and p38 MAPK can regulate the translocation of Bax to mitochondria, and promote the phosphorylation of Bcl-2 to accelerate its degradation, and subsequent activation of caspase-3 to lead to cell death [141,143]. Researchers have reported that upregulation of ALDH2 expression is accompanied by an increase in the myocardial Bcl-2/Bax ratio and a decrease in active caspase-3 levels, indicating that ALDH2 upregulation might help reduce the occurrence of myocardial-cell apoptosis [144].

Apoptosis of myocardial cells induced by OS is a main factor in the pathogenesis of heart failure. ALDH2 can protect myocardium by reducing myocardial-cell apoptosis caused by OS and oxidative damage to myocardium [145]. Studies suggest that ALDH2 can protect myocardial cells from OS and apoptosis induced by antimycin A by downregulating the MAPK signaling pathway [112]. In addition, ALDH2 can reduce renal cell apoptosis by inhibiting the MAPK pathway and increasing the Bcl-2/Bax ratio during ischemia/reperfusion (I/R) injury [146]. ALDH2 activated by Alda-1 has been shown to decrease the levels of MDA, 4-HNE, and cleaved caspase-3 expression, and increase the Bcl-2/Bax ratio in pancreatic acinar cells [147]. Overexpression of ALDH2 transgene prevents acetaldehyde-induced OS and inhibits the activation of key stress signaling molecules ERK1/2 and p38 MAPK as well as that of caspase-3, and also inhibits apoptosis of human umbilical-vein endothelial cells [70]. However, in the absence of ALDH2 protein from ALDH2 KO mice, expression of active caspases-9 and -3 in mouse myocardial cells is increased, which induces heart injury by activating ROS-dependent apoptosis as well as necroptosis medicated by receptor interacting protein kinases-1 and -3/mixed-lineage kinase domain-like protein (RIP1/RIP3/MLKL) [148]. It can be seen that the increase in ALDH2 expression is often accompanied by a reduction in MAPK signaling pathways or an increase in the Bcl-2/Bax ratio to reduce apoptosis [122,149,150]. Given the results of studies investigating the effect of MAPKs on the Bcl-2/Bax ratio, we speculate that ALDH2 inhibits apoptosis by increasing the Bcl-2/Bax ratio and reducing caspase-3 levels possibly via MAPK signaling pathway. However, the specific regulatory mechanism of each MAPK member on the Bcl-2/Bax ratio under the action of ALDH2 remains to be elucidated.

### 4.3. Aging

Evidence suggests that OS is a major underlying pathogenic factor in the aging process and related diseases. Cellular senescence is a stress response that links multiple pathologies, including atherosclerosis, stroke, and NDDs [151,152]. The general free-radical theory of aging attributes this process to the accumulation of damage from oxidative modifications of cellular components by ROS, which induces various diseases [153,154]. Some studies support a close relationship between ALDH2 deficiency and aging, especially cardiac and vascular diseases related to aging of ECs.

The activation of ALDH2 can increase SIRT1 levels to inhibit acetylation of the aging marker p53 and resist cell senescence. Studies have demonstrated that ALDH2 improves aging-related myocardial ischemic intolerance via a deacetylase sirtuin-dependent manner [155]. Sirtuin 1 (SIRT1) belongs to a class of NAD^+^-dependent histone deacetylases (sirtuins). It is widely involved in physiological activities such as fatty-acid oxidation and stress tolerance, and is closely related to apoptosis and aging [156,157]. Studies have found that activation or overexpression of ALDH2 can reduce levels of aging markers such as senescence-associated β-galactosidase (SA-β-gal) and p53/p21 in aging cells to reduce the risk of myocardial aging and atherosclerosis [158,159,160]. P53 is a downstream target of SIRT1 and plays a key role in cell aging [161,162]. ROS accumulated during OS can induce p53-dependent aging through the p38 MAPK and ataxia telangiectasia mutated protein (ATM) pathways. Meanwhile, 4-HNE produced by OS can combine with SIRT1 to form SIRT1–4-HNE adducts, which hinder the transfer of SIRT1 from cytoplasm to nucleus and reduce SIRT1 deacetylation activity, significantly increasing the level of acetylated p53 and promoting the occurrence of aging. ALDH2 affects the acetylation of p53 by regulating SIRT1 activity [158]. ALDH2 can also inactivate the EC p38 MAPK pathway and inhibit the ROS–MAPK–p53/p16 pathway to participate in the anti-aging process, as well as inhibit 4-HNE accumulation and protect endothelium by regulating SIRT1/p53-dependent EC senescence [120,161].

### 4.4. Nervous System Injury

Aldehydes produced by lipid peroxidation, especially 4-HNE, are related to nerve trauma, NDDs, and neuroinflammation [163]. The strong electrophilic properties of 4-HNE based on α,β-unsaturated structures make it highly reactive and liable to form stable protein adducts, causing cell damage [160,164]. Oxidative damage causes loss of nerve reserve and cognitive dysfunction. Alzheimer’s disease (AD) and Parkinson’s disease (PD) are both caused by progressive degeneration of the central nervous system (CNS).

OS is an important factor in the pathogenesis of AD. The main pathological characteristics of the AD brain are the presence of senile plaques (SPs) with abnormally aggregated β-amyloid (Aβ) as the main component, neurofibrillary tangles (NFTs) formed by hyperphosphorylated microtubule-associated protein tau, and neuronal loss. However, 4-HNE can significantly inhibit microtubule formation and neurite outgrowth and can react with phosphorylated tau to induce conformational changes in tau protein, thereby promoting NFT formation and causing neuronal death and synaptic dysfunction [12]. 4-HNE covalently modifies Aβ by 1,4 conjugates, altering Aβ’s function and impairing cell metabolism, signal transduction, and structural integrity [154]. Studies have demonstrated that the lack of ALDH2 activity caused by expression of the ALDH2*2 gene leads to accumulation of 4-HNE in the brains of transgenic mice and the death of neuronal cells, resulting in age-related neurodegeneration; this shows that ALDH2 can reduce 4-HNE–induced Aβ production and tau phosphorylation to prevent NFTs and SPs formation so as to inhibit neurotoxicity in AD [74].

The most prominent pathological changes in PD are the degeneration of dopaminergic neurons in the substantia nigra (SN) of the midbrain. The typical neuropathological feature is the aggregation of α-synuclein (α-Syn) in the remaining neurons into Lewy bodies (LBs) [165]. Studies have shown that 3,4-dihydroxyphenylacetaldehyde (DOPAL) is covalently modified with α-Syn by Lys residue, inducing the oligomerization of α-Syn and damaging synaptic vesicles of dopaminergic neurons [166]. ALDH2 either (1) catalyzes oxidation of the dopamine (DA) metabolite DOPAL to 3,4-dihydroxyphenylacetic acid (DOPAC), which can be further metabolized into homovanillic acid (HVA) or (2) catalyzes 3,4-dihydroxyphenylglycolaldehyde (DOPEGAL), the metabolite of norepinephrine (NE) and epinephrine (Epi), to 3,4-dihydroxymandelic acid (DOMA), which can be further metabolized into vanillylmandelic acid (VMA) so as to reduce ROS and caspase-3 protein expression as well as the aggregation of α-Syn to protect neurons from neurite damage [118,167,168]. That is, ALDH2 can inhibit α-Syn abnormal aggregation to form LBs in the SN of the brain by metabolizing the toxic DA metabolite DOPAL into DOPAC, thereby alleviating dopaminergic neuron injury in PD.

### 4.5. Other Oxidative Stress-Related Diseases

Studies have also demonstrated that ALDH2 deficiency is likely to cause the accumulation of aldehydes in the body under long-term adverse stimulation, which can induce gene mutations and tissue damage by reacting with biomolecules, such as proteins and nucleic acids, to generate various adducts. This increases the risk of hypertension, pulmonary hypertension and other vascular diseases, osteoporosis, and cancer [10,11,12,13,14,169] (Table 2). Among them, in addition to those on CCVDs, the mechanisms of ALDH2 involvement in cancer have been widely reported, including digestive system cancer, lung cancer, breast cancer, head and neck cancer [170,171,172]. ALDH2 is widely associated with cancer mediated by the metabolic disorder of alcohol and aldehydes, and it could be a possible prognostic biomarker for several types of cancer [173]. One of the most common studies is about the regulatory mechanism of ALDH2 gene polymorphism in digestive system cancer, such as esophageal cancer, colorectal cancer, gastric cancer, liver cancer, and pancreatic cancer [173,174,175,176,177]. ALDH2 was reported to protect against hepato-cellular carcinoma (HCC) metastasis by reducing acetaldehyde accumulation and activating the adenosine 5’-monophosphate-activated protein kinase (AMPK) pathway [169]. The expression of ALDH2 is decreased by silencing transcription factor FOXM1, which subsequently increases the level of Bax and cleaved-caspase-3 to promote the apoptosis of liver cancer stem cells (LCSCs) as well as inhibit tumorigenesis of LCSCs [178]. However, although there are many meta-analyses on the association between ALDH2 gene polymorphism and cancer risk, studies on the regulatory mechanism of ALDH2 involved in cancer pathogenesis and prognosis are still relatively scarce. ALDH2 plays an important role in the occurrence and development of OS-related diseases. Many diseases are accompanied by an increase in oxidation level or a decrease in antioxidant capacity to different degrees. The relationships between OS and the mechanisms of various diseases have received increasingly more attention [13], and research on the regulation of ALDH2 activity therefore deserves the same.

## 5. Regulation of ALDH2 Activity

### 5.1. Activators

Alda-1 is a specific agonist of ALDH2 [108,179,180,181,182]. It can activate WT ALDH2*1 and restore the activity of mutant ALDH2*2 to near-WT levels by acting as a structural chaperone. Alda-1 binds, not near the ALDH2*2 mutation site, but at the substrate entrance tunnel of the ALDH2 apo-enzyme, and extends to the active site. This mode of binding leaves the catalytic Cys302 and Glu268 residue unimpeded. After Alda-1 binds to ALDH2, dehydrogenase activity is activated by limiting substrate diffusion in the substrate-to-coenzyme tunnel, increasing the effective concentration of reactive groups within the active site. Alda-1 also reduces the accessibility of 4-HNE to Cys302 and its adjacent Cys301 and Cys303 residues and inhibits the formation of aldehyde adducts, reversing the inhibitory effect of 4-HNE on ALDH2 [176]. At the same time, the binding of Alda-1 restores the structures of the mutant ALDH2 (ALDH2*2) tetramer’s α-helix and active-site loop to near-similarity with those of their WT counterparts, improving structural stability and rescuing most of the functional defects [181,183].

Many studies have verified that Alda-1 is effective in activating ALDH2 activity. Some have found that Alda-1 improves brain injury and neurological function by increasing ALDH2 activity and reducing the accumulation of active aldehydes [184]. Alda-1, an activator of ALDH2, can significantly reverse the liver I/R injury of rats by inhibiting the production of ROS and reducing the accumulation of 4-HNE and MDA, all of which significantly decrease cell apoptosis and reduce liver pathological damage [185]. Another study found that levels of tumor necrosis factor-alpha (TNF-α) and IL-6 in the retinas of an Alda-1–treated group were lower; superoxide dismutase (SOD) activity was significantly higher; and expression of ALDH2, SIRT1, and Nrf2 was significantly increased. Meanwhile, expression of vascular endothelial growth factor-alpha (VEGF-α) was significantly decreased. These findings indicated that ALDH2 alleviates retinal damage [186]. In addition, Alda-1 can completely eliminate the increase in 4-HNE levels in neurons subjected to oxygen–glucose deprivation (OGD) and reduce neurotoxicity [37]. Other studies have found that Alda-1 treatment can reduce 4-HNE levels, reduce lung injury by activating ALDH2 activity, and improve the integrity of the lung epithelial barrier in rat models of severe hemorrhagic shock and resuscitation [187]. Alda-44 has also been shown to be a direct activator of ALDH2, which reduces I/R-induced cardiomyocytic apoptosis by decreasing the 4-HNE–mediated activation (phosphorylation) of stress-activated protein kinase (SAPK)/JNK [180]. Other studies have shown that activated protein kinase C epsilon (PKCε) can lead to activation of its downstream substrate ALDH2, which plays a protective role in heart and brain injury [37,85,88,188]. Moderate levels of ethanol [37,189], isoflurane [190], estrogen [140], heat shock factor 1 (HSF1) [85], and melatonin [188] have been reported to increase ALDH2 activity by enhancing the mitochondrial import of PKCε.

In addition to specific activators, antioxidants such as lipoic acid (LA) [191], resveratrol [192], and vitamins such as D and E indirectly activate ALDH2 activity via related signaling pathways, which can effectively reduce the risk of cell damage and disease. Previous studies showed that LA activated ALDH2 in animal models of heart failure, nitrate tolerance, and diabetes [193,194,195]. Consequently, these antioxidants and vitamins have been the subjects of further research and development [196,197].

### 5.2. Inhibitors

Daidzin, a kind of isoflavone that predominantly exists in glucoside form [198,199], is a specific inhibitor of ALDH2 in vitro. As with Alda-1, daidzin binds to ALDH2 at the entrance of the substrate tunnel, occupying a site that partially overlaps with Alda-1 [181]. However, the additional phenol arm of daidzin reaches the catalytic site and contacts Cys302 and Glu268, thereby blocking the catalytic function of ALDH2. Kinetic studies have demonstrated that daidzin inhibition is competitive against aldehyde substrates but not against NAD^+^ due to the considerable conformational flexibility of the latter when binding to ALDH2 [199].

Studies have shown that daidzin increases ROS production in mice with angiotensin II (Ang II)–induced hypertension by inactivating ALDH2, which increases vasoconstriction and aggravates hypertensive organ damage [200]. Other studies have found that daidzin’s inhibition of ALDH2 activity can significantly enhance apoptosis and ROS production in rat myocardial cells by activating the MAPK signaling pathway [112]. In addition, daidzin can promote differentiation of HCFs induced by TGF-β1 and the fibrotic process in myocardial cells [111,128].

Cyanamide (CYA) [37,201], disulfiram (DSF) [199,201,202], and other non-specific inhibitors can also indirectly inhibit ALDH2 activity through related signaling pathways, giving them application value in both clinical treatment of diseases and scientific research. Studies have demonstrated that CYA can downregulate expression of ALDH2 protein in a rat acute kidney injury (AKI) model induced by cecal ligation and puncture (CLP). Researchers have found that levels of plasma creatinine (CRE), blood urea nitrogen (BUN), renal MDA, and NF-κB p65 protein expression increased while SOD activity decreased and glomerular atrophy was aggravated, indicating that CYA aggravates renal injury by inhibiting ALDH2 [121]. DSF, which was approved by the FDA to treat alcohol abuse and dependence in 1951 [203], is bioactivated to S-methyl N, N-diethylthiocarbamate sulfoxide (DETC-MeSO) to inhibit ALDH2. ALDH2 inhibition by DSF exacerbates the Ang II–induced decrease in coronary angiogenesis by lowering levels of VEGF receptors (VEGFR1 and VEGFR2) and 4-HNE adducts to ameliorate cardiometabolic diseases [204]. DSF can also be used in aversion therapy for alcohol abuse and dependence; it increases acetaldehyde in the blood and leads to disulfiram–alcohol reactions such as nausea, vomiting, headache, hypotension, and tachycardia, which deter patients from consuming alcohol.

### 5.3. Post-Translational Modifications (PTMs) of ALDH2

PTMs of some mitochondrial enzymes are involved in the regulation of ROS production and oxidative metabolism, thereby playing important roles in cellular functions. Different types of protein PTMs have different effects on ALDH2 activity [205].

PKCε phosphorylation is important in the regulation and protection of ALDH2 enzyme activity. PKCε can activate ALDH2 by inducing phosphorylation of serine/threonine in ALDH2, while PKCε can be activated by several pathways [88]. Studies have shown that ethanol can activate PKCε during I/R in vivo [180,206] and ALDH2 is further activated by phosphorylation of Thr^185^, Thr^412^, and possibly Ser^279^ to protect the heart [179]. Estrogen in females can also lead to ALDH2 phosphorylation by activating phosphatidylinositol 3-kinase (PI3K) and PKC so as to reduce production of ROS and toxic aldehydes; this might be why the degree of I/R injury is lower in female than in male rats [207]. Research has found that PKCε is activated after I/R and accelerates its transfer to mitochondria under the action of Hsp90, increasing the phosphorylation and activity of ALDH2 [208]. In addition, isoflurane, a volatile anesthetic, can mediate ALDH2 phosphorylation and protect the heart from I/R injury through PKCε translocation from cytoplasm to mitochondria [190]. Under different mechanisms, phosphorylation at different sites also inhibits ALDH2 enzyme activity. In contrast to PKCε-mediated phosphorylation and subsequent activation of ALDH2, one study indicated that CCl_4_ exposure activates JNK, which translocates to mitochondria and, there, phosphorylates and inhibits ALDH2 [209].

SIRT3, which is mainly located in the mitochondrial matrix, is a histone deacetylase that depends on NAD^+^ to regulate the acetylation level of lysine residues [88,205]. Lysines of ALDH2 are also targets of deacetylase SIRT3 [210]. SIRT3 can change the acetylation level of lysine sites in the domain that ALDH2 binds with its cofactor NAD^+^, thereby affecting the binding of these sites; acetylation changes the structure of homotetramer and affects its activity. However, reports differ on acetylation’s effect on ALDH2 activity. Most studies suggest that SIRT3-mediated deacetylation reduces the activity of ALDH2 dehydrogenase [16,88]. Some research has shown that under acute ethanol exposure, SIRT3 is inactivated due to the decrease in NAD^+^/NADH ratio induced by low-dose ethanol, leading to high acetylation and activation of ALDH2 [105,211]. Other studies have found that acetaminophen (N-acetyl-p-aminophenol [APAP]) promotes SIRT3-mediated ALDH2 deacetylation and deactivation [212]. Conversely, acetylation of ALDH2 in vitro leads to a significant decrease in enzymatic activity, while SIRT3 can partially restore ALDH2 activity [210]; this study was conducted completely in vitro using human recombinant ALDH2 protein, which cannot completely simulate the complex living environment of cells in vivo, but those findings also suggested that regulation of acetylation by ALDH2 activity involves a more complex mechanism, which is worthy of further exploration.

ALDH2 activity can be inhibited by such conditions or substances as chronic or excessive ethanol abuse [213,214], APAP [215], nitric oxide (NO) donor [215], and I/R injury [216] via cysteine S-nitrosylation and tyrosine nitration, because of the increase in the production of peroxynitrite (ONOO^−^) [217]. Mitochondria-targeted lipophilic ubiquinone (MitoQ) can prevent alcohol-induced S-nitrosylation of cysteine residues and nitration of tyrosine residues from impairing ALDH2 activity, so as to restore ALDH activity, and reduce alcohol-induced liver and intestinal damage [214].

**Figure 4:**
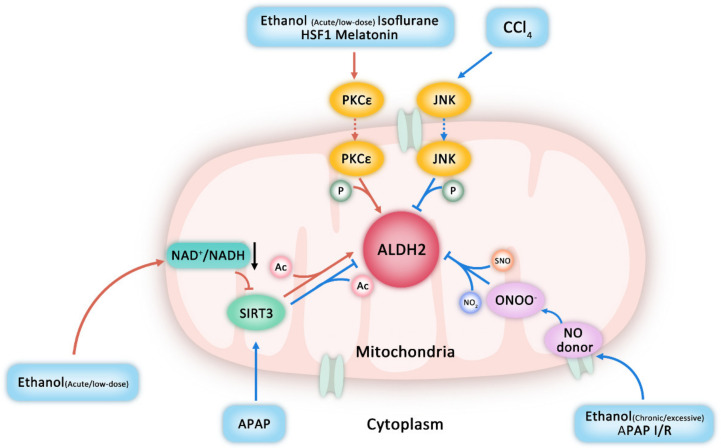
Effect of protein post-translational modifications (PTMs) on ALDH activity. Ethanol (acute/low-dose), isoflurane, heat shock factor 1 (HSF1), and melatonin can lead to phosphorylation and activation of aldehyde dehydrogenase 2 (ALDH2) by activating phosphatidylinositol protein kinase C epsilon (PKCε). ALDH2 can also be activated by its acetylation increased by the inactivation of sirtuin 3 (SIRT3) stimulated by ethanol (acute/low-dose). The phosphorylation of ALDH2 promoted by c-Jun NH_2_-terminal kinase (JNK) under carbon tetrachloride (CCl_4_) exposure as well as the S-nitrosylation and tyrosine nitration of ALDH2 stimulated by ethanol (chronic or excessive abuse), acetaminophen (N-acetyl-p-aminophenol [APAP]), nitric oxide (NO) donor, and ischemia/reperfusion (I/R) injury can inhibit ALDH2 activity. P = phosphorylation; Ac = acetylation; SNO = S-nitrosylation; NO_2_ = nitration.

In the aggregate (Figure 4), phosphorylation is generally related to activation of ALDH2 enzyme activity, but phosphorylation through the JNK pathway leads to inactivation of ALDH2. Most studies have revealed the promotional effect of acetylation on ALDH2 enzyme activity, but this must be confirmed by additional studies. The more consistent conclusion is that S-nitrosylation and nitration generally cause a decrease in ALDH2 enzyme activity. In addition, ubiquitination and glycosylation are widely involved in the regulation of OS protein function in vivo, but few studies have been conducted on their relationship with the regulation of ALDH2 activity. There is a synergistic and competitive relationship between different modification types, such as the mutual stimulation and inhibition among phosphorylation, methylation, glycation, acetylation, and ubiquitination [218,219,220,221], which plays an important role in the function of the target protein. Moreover, different modifications play different roles such as activation, inhibition, and even degradation of the target protein (such as ubiquitination) [222,223,224,225,226]. The synergy or inhibition among different modifications, together with their impact on the activity of the target protein, is closely related to the types of target proteins and modification sites of the modification, which deserves more attention in follow-up research.

## 6. Conclusions

ALDH2 not only plays an important role in the prevention and treatment of alcohol abuse, but also widely participates in the key regulatory pathways of diseases related to apoptosis, aging, fibrosis, and neurodegeneration [105]. A certain degree of research on the activation of ALDH2 and improvement of its function, in particular the compensation for the functional defects of its mutant genotypes (ALDH2*1/2, ALDH2*2/2), has been carried out. Alda-1, an effective activator of ALDH2, has certain clinical value and application potentialities, but due to its poor solubility in most common solvents, its physical and chemical properties still need to be further explored to improve the drug-like properties. In addition to specific activators, other non-specific activators, such as antioxidant LA can indirectly activate ALDH2 through related signaling pathways and inhibit toxic aldehydes caused by OS and cytotoxicity. LA is an over-the-counter supplement that acts as a potent antioxidant and has been widely used as supplementary treatment of diabetic neuropathy [227,228]. However, further extensive and in-depth exploration of the regulation of non-specific activators on ALDH2 and related signaling pathway is still needed.

When exploring the regulation mechanism of ALDH2 activity, the role of PTMs cannot be ignored. ALDH isozymes can be modified by various forms of PTMs, including phosphorylation, acetylation, and S-nitrosylation, which affect its activity effectively [154]. Although most studies show that phosphorylation and acetylation are mainly involved in the activation mechanism of ALDH2 activity, nitrosylation and nitrification mainly damage its activity, it is not excluded that sometimes different upstream regulatory substances or different modification sites will also have the opposite effect. Regulation of ALDH2 by other PTMs and the synergy or inhibition between different types of PTMs are worthy of further research. Therefore, in addition to the exploration of safer and more efficient activators of ALDH2 and their regulatory pathways, the degree and regulatory mechanisms of its key regulatory PTMs and the interaction among different modification types are also worthy of attention. In the context of human diseases associated with ALDH2 activity, it will be worth exploring the roles played by PTMs of ALDH2 and their related regulatory pathway in the future.

## Figures and Tables

**Table 1:** Human aldehyde dehydrogenase (ALDH) gene superfamily.

Gene Name	Chromosomal Location	Protein Subcellular Location	Expression	Substrate/Ref.
*ALDH1A1*	9q21.13	Cytoplasm, cytosol	Liver, duodenum, 18 other tissues	Retinaldehyde [19,20,21]; Acetaldehyde [22]
*ALDH1A2*	15q21.3	Cytoplasm, cytosol	Endometrium, testes, 8 other tissues	Retinaldehyde [23,24,25]
*ALDH1A3*	15q26.3	Cytoplasm, cytosol	Prostate, bladder, 14 other tissues	Retinaldehyde [26,27]
*ALDH1B1*	9p13.1	Mitochondrion	Liver, kidneys, 19 other tissues	Acetaldehyde [28,29]
*ALDH1L1*	3q21.3	Cytoplasm, cytosol	Liver, kidneys, 8 other tissues	10-Formyltetrahydrofolate [30,31,32]
*ALDH1L2*	12q23.3	Mitochondrion	Pancreas, salivary gland, 22 other tissues	10-Formyltetrahydrofolate [33,34]
*ALDH2*	12q24.12	Mitochondrion	Fat, liver, 19 other tissues	Acetaldehyde [35,36]; 4-Hydroxy-2-nonenal (4-HNE) [37,38]; Malondialdehyde (MDA) [39]
*ALDH3A1*	17p11.2	Cytoplasm, cytosol	Esophagus, stomach, skin	Benzaldehyde [36,40,41]
*ALDH3A2*	17p11.2	Endoplasmic reticulum	Skin, adrenal glands, 22 other tissues	Hexadecenal [42,43,44]
*ALDH3B1*	11q13.2	Plasma membrane	Lungs, bone marrow, 17 other tissues	Octaldehyde [36]; Benzaldehyde [45]; 4-HNE [45,46]; Hexanal [45,46]; Nonanal [46]
*ALDH3B2*	11q13.2	Lipid droplet	Skin, esophagus, 3 other tissues	Medium-chain to long-chain aldehydes [47]
*ALDH4A1*	1p36.13	Mitochondrion	Kidneys, liver, 8 other tissues	Glutamate γ-semialdehyde [48,49,50,51]
*ALDH5A1*	6p22.3	Mitochondrion	Liver, brain, 23 other tissues	Succinic semialdehyde [52,53]; 4-HNE [53]
*ALDH6A1*	14q24.3	Mitochondrion	Kidneys, liver, 11 other tissues	Methylmalonate-semialdehyde [54]
*ALDH7A1*	5q23.2	Cytoplasm, cytosol, mitochondrion, nucleus	Kidneys, liver, 24 other tissues	α-Aminoadipic-semialdehyde [55,56]
*ALDH8A1*	6q23.3	Cytoplasm, cytosol	Liver, kidneys	Retinaldehyde [36,57]
*ALDH9A1*	1q24.1	Cytoplasm, cytosol	Fat, thyroid gland, 25 other tissues	γ-Trimethylaminobutyraldehyde [58,59]
*ALDH16A1*	19q13.33	Membrane	Spleen, duodenum, 25 other tissues	Unknown, lacks measurable catalytic activity [60]
*ALDH18A1*	10q24.1	Mitochondrion	Duodenum, small intestine, 25 other tissues	Glutamate; γ-Glutamyl phosphate [61,62,63,64]

Basic gene information was acquired from the NCBI (National Center for Biotechnology Information) and Uniprot (Universal Protein) databases.

**Table 2:** Function and regulatory pathways of ALDH2 in oxidative stress-related diseases and injury.

Organ	Treatment	Tissue/Cell, Species	Disease/ Injury	Molecular Mechanism	ALDH2 Function	Notes	Ref.
Heart	ALDH2 (indirectly activated)	Human cardiac fibroblasts (HCFs), human	Myocardial fibrosis	(−) TGF-β1, Smad → (+) ALDH2 → (−) α-SMA, myofibroblastic proliferation, collagen	Inhibit myocardial fibrosis induced by TGF-β1	TGF-β1: transforming growth factor-β1 ALDH: aldehyde dehydrogenase α-SMA: α-smooth muscle actin Smad: *Drosophila* mothers against decapentaplegic protein	[111]
	ALDH2 (inhibited by daidzin)	Heart, rat	Cardiomyocytic apoptosis	Daidzin → (−) ALDH2 → (+) ERK1/2, JNK, p38 MAPK → (+) ROS, 4-HNE → (+) cardiomyocytic apoptosis	Inhibit cardiomyocytic apoptosis by downregulating the MAPK pathway	ERK1/2: extracellular signal–regulated kinase 1/2 JNK: c-Jun NH_2_-terminal kinase p38 MAPK: p38 mitogen-activated protein kinase 4-HNE: 4-hydroxy-2-nonenal ROS: reactive oxygen species	[112]
	ALDH2 (activated by RIPostC)	Heart, rat	Myocardial ischemia/reperfusion (I/R) injury	RIPostC → (+) ALDH2, Bcl-2/Bax ↑ → p-Akt/Akt ↑ → (−) cleaved caspase-3 → (−) cardiomyocytic apoptosis	Help mediate the cardio-protection of RIPostC via the PI3K—Akt pathway	RIPostC: remote ischemic postconditioning Bcl-2: B-cell lymphoma-2 Bax: Bcl-2–associated X protein Akt: protein kinase B PI3K: phosphatidylinositol 3-kinase caspase-3: cysteinyl aspartate–specific proteinase 3	[113]
Liver	*ALDH2*^−^^/^^−^ (KO)	Liver, mouse	Alcoholic liver injury	Alcohol, CCl_4_, *ALDH2*^−^^/^^−^ → (+) MAAs, acetaldehyde → (+) IL-6 → (+) ERK1/2, p38 MAPK, STAT3, TGF-β, α-SMA, COLIA1, TIMP-1 → liver inflammation, fibrosis	*ALDH2* deficiency accelerates alcohol-induced liver inflammatory response and fibrosis	CCl_4_: carbon tetrachloride MAAs: malondialdehyde–acetaldehyde adducts IL-6: interleukin-6 STAT3: signal transducer and activator of transcription 3 COLIA1: collagen type I alpha 1 TIMP-1: tissue inhibitor of metalloproteinase-1	[114]
	*ALDH2*^−^^/^^−^ (KO)	Liver, mouse	Chronic liver fibrosis	CCl_4_, *ALDH2*^−^^/^^−^ → (+) TGF-β1, ROS → Bcl-2/Bax ↓, Nrf2/HO-1 ↓; (+) TIMP-1, p62, α-SMA, COLIA1; (−) Parkin → (+) fibrosis	Alleviate CCl_4_-induced hepatic fibrosis via Nrf2/HO-1 pathway	Nrf2: nuclear factor erythroid 2-related factor 2 HO-1: heme oxygenase-1	[115]
Nerve	*ALDH2*^−/−^ (KO)	Brain, mouse	Alzheimer disease (AD)	*ALDH2*^−/−^ → (+) 4-HNE → (+) p-tau, Aβ, activated caspases → (+) NFTs → synaptic and mitochondrial dysfunction → neurodegeneration	Defects in ALDH2 activity kill neurons by stimulating the accumulation of 4-HNE due to OS	tau: microtubule-associated protein tau NFTs: neurofibrillary tangles Aβ: β-amyloid	[116,117]
	ALDH2	Pheochromocytoma (PC12), rat	Parkinson disease (PD)	DA, NE, EPI → DOPAL, DOPEGAL + ALDH2 → DOPAC, DOMA → HVA, VMA, (−) α-Syn, (−) LBs → (−) neurotoxicity	Promote removal of neurotoxic metabolites produced in the metabolism of monoamine neurotransmitters and reduce neurotoxicity	DA: dopamine NE: norepinephrine EPI: epinephrine DOPAL: 3,4-dihydroxyphenylacetaldehyde DOPEGAL: 3,4-dihydroxyphenylglycolaldehyde DOPAC: 3,4-dihydroxyphenylacetic acid DOMA: 3,4-dihydroxymandelic acid HVA: homovanillic acid VMA: vanillylmandelic acid α-Syn: α-synuclein LBs: Lewy bodies	[118,119]
Blood vessel	ALDH2 (transgenic over-expression)	Human umbilical-vein endothelial cells (HUVECs), human	Apoptosis in HUVECs	Alcohol, (+) ALDH2 → (−) ROS, ERK1/2, p38 MAPK, caspase-3 → (−) HUVEC apoptosis	Alleviate OS and apoptosis of HUVECs under acetaldehyde exposure via MAPK pathway	−	[70]
	*ALDH2*^−^^/^^−^ (KO)	Human aorta epithelial cells (HAECs), human	Endothelial senescence	*ALDH2*^−^^/^^−^ → (+) 4-HNE → (−) SIRT1 → (−) p53 deacetylation → (+) endothelial senescence	Promote SIRT1 nuclear translocation by inhibiting 4-HNE accumulation to alleviate senescence	SIRT1: sirtuin 1	[120]
Others	ALDH2 (indirectly activated)	Kidney, rat	Acute kidney injury (AKI)	CYA → (−) ALDH2, SOD; (+) plasma CRE, BUN, MDA, p65, NF-κB → aggravate glomerular atrophy	Inhibition of ALDH2 aggravated the renal injury	CYA: cyanamide CRE: creatinine BUN: blood urea nitrogen MDA: malondialdehyde SOD: superoxide dismutase	[121]
	ALDH2 (activated by Alda-1)	Intestine, mouse	Intestinal I/R injury	Alda-1 → (+) ALDH2 → (−) 4-HNE, MDA, MPO, NO, iNOS, H_2_O_2_, caspase-3, NF-κBα, TNF-α, IL-6, IL-1β; (+) IκBα, Bcl-2/Bax ↑ → alleviate inflammatory response, OS	ALDH2 activation can alleviate intestinal I/R injury by relieving inflammatory response and OS	Alda-1: N-(1,3-benzodioxol-5-ylmethyl)-2,6-dichlorobenzamide MPO: myeloperoxidase iNOS: inducible NO synthase H_2_O_2_: hydrogen peroxide NF-κB: nuclear factor κ-light-chain-enhancer of activated B cells TNF-α: tumor necrosis factor-alpha IκBα: inhibitor of NF-κB IL-1β: interleukin-1β	[122]
	ALDH2*2 (transfected with the ALDH2*2 gene)	Osteoblasts, mouse	Osteoporosis	ALDH2*2 → (+) 4-HNE, acetaldehyde → (+) PPARγ → (−) osteoblastic differentiation → (+) osteoblastic apoptosis → osteoporosis	ALDH2*2 as a dominant-negative form of *ALDH2* promotes osteoporosis by impairing osteoblastogenesis	PPARγ: peroxisome proliferator–activated receptor gamma	[10]

(+) = activation/upregulation/*ALDH2* overexpression; (−) = inhibition/downregulation; ^−/−^ = knockout/deletion; KO = knockout; ↑ = increase of ratio; ↓ = decrease of ratio; p- = phosphorylation; A + B = substance A interacts with substance B.
